# Mathematical Modelling of the MAP Kinase Pathway Using Proteomic Datasets

**DOI:** 10.1371/journal.pone.0042230

**Published:** 2012-08-08

**Authors:** Tianhai Tian, Jiangning Song

**Affiliations:** 1 School of Mathematical Sciences, Faculty of Science, Monash University, Clayton, Victoria, Australia; 2 Department of Biochemistry and Molecular Biology, Faculty of Medicine, Monash University, Clayton, Victoria, Australia; Memorial Sloan Kettering Cancer Center, United States of America

## Abstract

The advances in proteomics technologies offer an unprecedented opportunity and valuable resources to understand how living organisms execute necessary functions at systems levels. However, little work has been done up to date to utilize the highly accurate spatio-temporal dynamic proteome data generated by phosphoprotemics for mathematical modeling of complex cell signaling pathways. This work proposed a novel computational framework to develop mathematical models based on proteomic datasets. Using the MAP kinase pathway as the test system, we developed a mathematical model including the cytosolic and nuclear subsystems; and applied the genetic algorithm to infer unknown model parameters. Robustness property of the mathematical model was used as a criterion to select the appropriate rate constants from the estimated candidates. Quantitative information regarding the absolute protein concentrations was used to refine the mathematical model. We have demonstrated that the incorporation of more experimental data could significantly enhance both the simulation accuracy and robustness property of the proposed model. In addition, we used the MAP kinase pathway inhibited by phosphatases with different concentrations to predict the signal output influenced by different cellular conditions. Our predictions are in good agreement with the experimental observations when the MAP kinase pathway was inhibited by phosphatase PP2A and MKP3. The successful application of the proposed modeling framework to the MAP kinase pathway suggests that our method is very promising for developing accurate mathematical models and yielding insights into the regulatory mechanisms of complex cell signaling pathways.

## Introduction

In the post-genomic era, proteomics is considered as the next crucial step to study biological systems because it allows large-scale determination of genetic and cellular functions at the protein level [1], [2]. The proteome is the entire complement of proteins, including the post-translational modifications (PTMs) that are made to a particular set of proteins. Unlike the genome that is more or less constant, the proteome differs from cell to cell, as well as varies over time and distinct requirements that a cell or organism undergoes [3]. The purpose of proteomics research is to determine the relative or absolute amount of a biological sample. In recent years, the advanced proteomic technologies, including mass spectrometry (MS), two-dimensional gel electrophoresis and protein arrays, provide powerful methods for analyzing protein samples, emerging as a potent tool for rapidly identifying proteins from complex biological samples, and for characterizing protein post-translational modifications and protein-protein interactions [4], [5].

An important application of MS-based proteomics is to study cell signaling cascades that involve the binding of extracellular signaling molecules to cell-surface receptors triggering events inside the cell [6]. In this process, phosphorylation, a key reversible PTM, plays a key role in regulating protein function and localization in cell signaling networks. Phosphoproteomics is a branch of proteomics that identifies and characterizes proteins containing a phosphate group as a PTM [6], [7]. In recent years phosphoproteome studies have provided a global and integrative description of cellular signaling networks [8], [9], [10], [11]. However, the complex nature of the cell signaling pathways remains to be completely understood as to how they are exactly regulated *in vivo* and what are the important parameters that determine their dynamics [12]. In this context, mathematical modeling is a powerful tool for addressing such key questions, deducing useful regulatory principles and understanding the complex biological systems [13]. To improve our understanding of signaling pathways, mathematical modeling allows us to make testable predictions and validate biological hypotheses regarding the signal transduction mechanisms regulating various cellular functions [14].

One of the major challenges in systems biology is the lack of kinetic rates for mathematical modeling that ideally should be measured by experiments or estimated from experimental data. Although mathematical models have been developed to study various cell signaling pathways, these models were predominantly designed based on either *in vitro* assays or in-cell Western blot assays. Due to the limited amount of experimental data, a common approach currently used in systems biology is to collect published experimental data that were obtained from different cell types under various conditions. Therefore the advances in proteomics technologies offer an unprecedented opportunity to understand how living organisms execute necessary functions at systems levels. From a systems biology perspective, the highly accurate temporal dynamic data generated by phosphoprotemics are valuable resources to infer unknown model parameters and to accurately model complex cell signaling networks. However, little work has been done up to date to utilize the temporal dynamic proteome dataset in mathematical modeling of biological systems. Although it was claimed that the proteomic data were used in a number of research works [15], [16], [17], these studies in fact still heavily relied on traditional experimental data such as Western blot assays. However, experimental studies have recently expedited the research to generate proteomic data for inferring kinetic parameters and developing more accurate signaling pathway models [18], [19], [20], [21].

One of the most prominent signaling pathways, the mitogen-activated protein (MAP) kinase cascade, communicates signal from the growth factor receptors on the cell surface to effector molecules located in the cytoplasm and nucleus. This pathway is activated by the upstream input signal Ras protein, and comprises a set of three protein kinases, namely Raf, MEK and ERK, with a highly conserved molecular architecture that acts sequentially [22]. Activated MAP kinase phosphorylates multiple substrates, including transcription factors, protein kinases, phospholipases and cytoskeletal proteins, as well as regulates a wide range of physiological responses, such as cell proliferation, differentiation, apoptosis, and tissue development. Note that the signaling downstream of Ras protein is of an incredible complexity that includes positive and negative feedback loops, protein re-localization, signaling complex formation and cross-talk between parallel signaling pathways. The EGF-regulated MAP kinase pathway is among the best-characterized signal transduction pathways. Although the principal hierarchy of the signaling pathway and its activation sequence are well established, recent experimental data have yielded additional information on critical protein-protein interactions, regulatory loops and spatio-temporal organization [23].

Over the last decade, the MAP kinase pathway has been used repeatedly as a testable paradigm for pioneering computational systems biology. By focusing on Ras-dependent activation of the MAP kinase module, Huang and Ferrell developed the first mathematical model that predicted highly ultra-sensitive responses of the MAP kinase cascade, which were then confirmed by experimentation [24]. The success of this work stimulated a great deal of interests in designing kinetic models that provided testable predictions and novel insights into signaling events. For example, Bhalla et al. combined experiments and modeling to support MAP kinase involvement in a bistable feedback loop [25]; Schoeberl et al. developed the mathematical model for the EGF-regulated MAP kinase pathway [26]; we have demonstrated that the critical function of Ras nanoclusters in generating high-fidelity signal transduction [27]; and recent research works investigated the cross-talk between the MAP kinase pathway and other parallel signaling pathways [28]. Nevertheless, the molecular mechanisms that allow for precise yet robust control of MAP kinase signal intensity with a range of activation kinetics and diverse biological outcomes remain poorly understood. Using the MAP kinase pathway as the test system, this work will design a novel computational framework for developing mathematical models of cell signaling pathway based on the available proteomic data, which represents one of the earliest effects in using proteomic data to develop detailed mathematical models.

## Results

### Development of mathematical model

**Figure 1:**
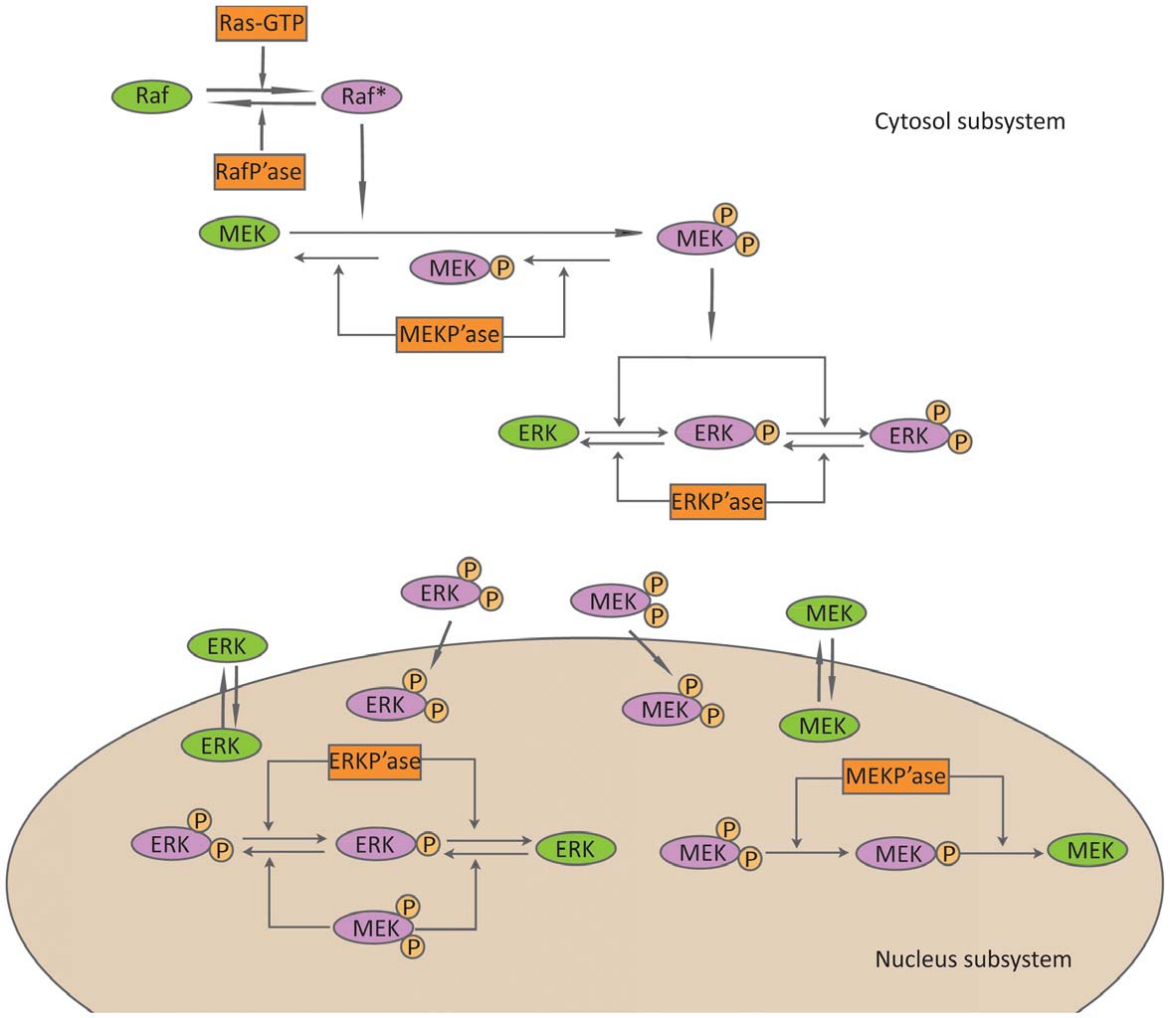
Schematic representation of the MAK kinase pathway.

Our proposed model of the MAP kinase pathway comprises a cytosolic subsystem and a nuclear subsystem (Fig. 1). In the cytosolic subsystem, the Ras-GTP is the signal input of the MAP kinase cascade, which activates Raf molecules in a single step. This activation is followed by sequential activation of the dual-specificity MAP kinase kinase (MEK) by Raf* (i.e., the activated Raf) in a single-step processive module. The activated MEKpp (i.e. phosphorylated MEK at two residue positions) in turn activates ERK in a two-step distributive module [29]. The activated ERKpp (i.e. phosphorylated ERK at two residue positions) is the signal output of the MAK kinase module. Both the activated and un-activated MEK and ERK kinases can diffuse between the cytosol and nucleus freely. In the nuclear subsystem, the activated MEKpp can further activate ERK kinase via the distributive two-step phosphorylation module. In addition, phosphatases, termed as Raf-P'ase, MEK-P'ase and ERK-P'ase, can deactivate the activated Raf*, MEKpp and ERKpp kinases, respectively, at different subcellular locations.

A set of chemical reactions was used to describe the detailed process of kinase activation. Briefly, the activated kinase (or phosphatase) K binds to its substrate S (or activated kinase Sp) to form a protein complex K-S (or K-Sp), which leads to the activated substrate Sp (or deactivated kinase S). Examples of these reactions are: the processive phosphorylation module of MEK kinase
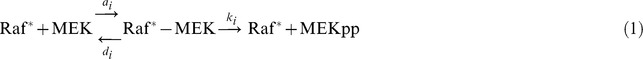
and the distributive phosphorylation module of ERK kinase
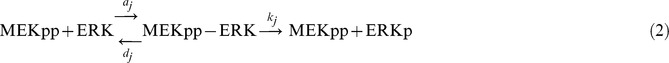


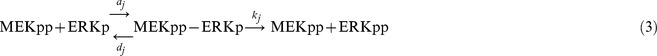
where 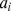, 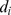 and 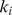 are protein binding, dissociation and activation rate constants, respectively. In addition, the diffusion of MEK kinase, for example, between the cytosolic and nuclear subsystems is represented by
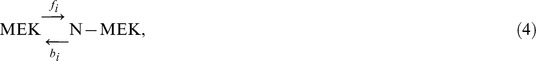
where MEK and N-MEK are MEK kinase located in the cytosolic and nuclear subsystems, respectively, 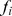 and 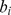 are diffusion rate constants. All the chemical reactions are listed in Supporting Information S1.

A mathematical model was developed according to the chemical rate equations of these chemical reactions. For example, reaction (1) leads to the following differential equation for the dynamics of the Raf*-MEK complex, given by
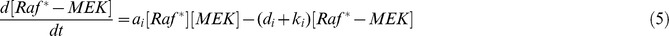
This mathematical model includes 33 differential equations representing the dynamics of 33 variables in the system. To test all the possibilities of the molecular mechanisms, we did not make any assumptions regarding the model rate constants and thus there are 57 unknown reaction rate constants. Detailed information of the differential equations is given in Supporting Information S1.

### Estimation of model kinetic rates

We first used the genetic algorithm to infer the model kinetic rates based on the proteomic dataset [9]. The corresponding model was termed as System 1. Since Ras activity was not available in this dataset, we used the Ras activity monitored *in vivo* by FRET imaging as the signal input of the MAP kinase module [30]. It was assumed that the total concentration of each kinase or phosphatase was unit one. The initial condition of the differential equation model was given in Table 1. Since the kinase activities in the proteomic dataset were available at most five time points, we used the linear interpolation to generate kinase activities at other 16 time points during the time interval [0,20] (min). To be consistent with the normalized kinase activities in the proteomic dataset [9], the simulated activity of each kinase was also normalized by its activity at 5 min; and we chose 
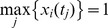
 in the objective function (6) for calculating the error between the simulation and proteomic data. The number of the unknown rate constants in the proposed model is 57. The parameter set that produced smaller simulation error with respect to the proteomics data was selected as the estimated model rate constants. In the genetic algorithm, the searching range of [0, W_max_] for each rate constant is the same (W_max_ = 1000). Because of the local maximal issue of the genetic algorithm, we implemented the genetic algorithm with different random seeds that led to different estimates of the model kinetic rates. We obtained 20 sets of estimated rate constants and selected the top 10 estimates with smaller simulation errors to the proteomic data for further analysis. The difference between the simulation errors of these top 10 estimates is quite small. It is thus reasonable to use any set of the estimated rate constants as the final estimate. Here we used the robustness property of the model as an additional criterion to select the optimal rate constants.

**Table 1:** Protein concentrations of the pathway models.

	Initial condition of System 1	Initial condition of Systems 2	Max % of activated kinase at 5 min in System 2	Activated kinases at 5 min in System 2
[Ras]	1	0.4 [30]		0.4
[Raf]	1	0.013 [30]		0.013
[Raf-P'ase]	1	0.002 [26]		
[MEK]	1	1.4 [30]	5% [30]	0.07
[MEK-P'ase]	1	0.14 [26]		
[ERK]	1	0.96 [30]	50% [30]	0.48
[ERK-P'ase]	1	0.48 [26]		

System 1 is the model based on the proteomic data only with normalized protein concentrations. System 2 is the model based on both proteomic and other experimental data with absolute protein concentrations. Except the variables in this table, the initial conditions of other variables are zeros. The concentrations of three phosphatases were calculated based on both the absolute kinase concentration in [30] and ratio of phosphatase concentration to the corresponding kinase concentration in [26].

Robustness, in both biological and engineering systems, can be defined as the ability of a system to function correctly in the presence of both internal and external uncertainty [31]. First introduced by Csete and Doyle [32], this theory has been extensively studied by Kitano and co-workers [33], [34], [35], [36]. Since robustness is a ubiquitously observed property of biological systems [33], [37], this property has been widely used recently as an important measure to select the optimal network structure or model rate constants from estimated candidates, including the MAP kinase pathway [38], [39], [40]. A formal and abstract definition of the robustness property, given by Kitano [34], is well consistent with the general principle of the robustness property of complex systems [31], and has been widely used in analyzing robustness properties of biological systems. Recently more detailed definitions have been proposed to calculate the robustness property of biological systems [41].

**Figure 2:**
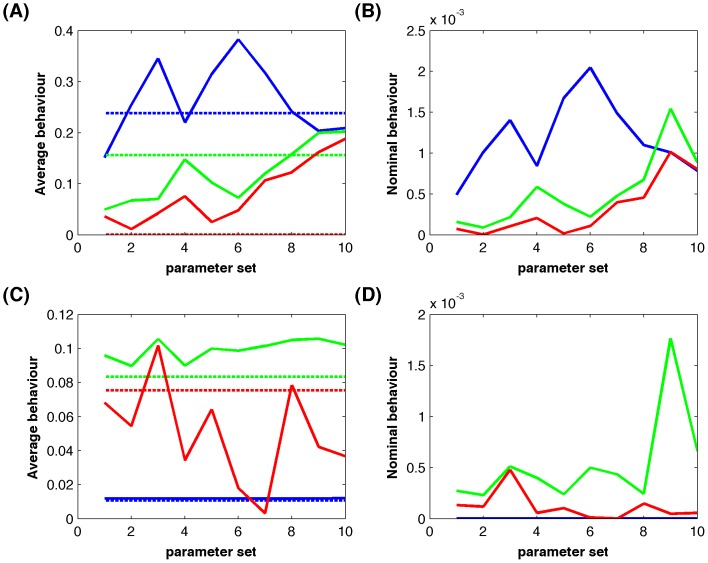
Robustness analysis. (A and B) Robustness analysis of the proposed model with 10 sets of estimated kinetic rates that were derived from the normalized proteomic data. (A) the average behavior and (B) nominal behavior of the model with perturbed kinetic rates. (C and D) Robustness analysis of the proposed model with 10 sets of estimated kinetic rates that were derived from more resources of experimental data. (C) the average behavior and (D) nominal behavior of the model with perturbed kinetic rates. (Blue-line: Raf; green-line: MEK, red-line: ERK. The horizontal dash lines in (A) and (C) are the simulated kinase activities based on the unperturbed model kinetic rates).

To choose the best set of kinetic rates, we then carried out the robustness analysis of the mathematical model for the selected 10 estimates of kinetic rates. We first used the estimated kinetic rates without any perturbation to produce a simulation that was used as the standard kinase activity. Then for each set of model rate constants, we perturbed the value of each parameter by using the generated random number. New simulations were obtained by using the perturbed rate constants, and we compared the new simulations with the standard simulation derived from the unperturbed model rate constants. The system with a particular set of rate constants is more stable if the difference between the new simulations and standard simulation is smaller. For each set of estimated rate constants, we generated 10,000 sets of perturbed rate constants by using the uniformly distributed random variable and 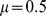 in equation (10). To make a fair comparison, the same random numbers in either the uniformly distributed random variable (10) or standard Gaussian random variable (11) were used in each set of rate estimate. The kinase activities at different subcellular locations together with the total activities of each kinase were collected at 20 min and we calculated the mean and variance of each kinase activity. Based on Kitano's definition of robustness [34], in this work we proposed to use the average behavior, which is the sum of all the means of each kinase activity as calculated by equation (8), and the nominal behavior, which is the sum of all the variances of each kinase activity as calculated by equation (9), as the measure of the robustness property. A model is more stable in terms of the average behavior if the perturbed behavior (solid lines in Figures 2A and 2C) is closer to the unperturbed behavior (dash-lines in Figures 2A and 2C). However, a model is more stable in terms of the nominal behavior if the values of the nominal behavior in Figures 2B and 2D are smaller.

Figures 2A and 2B give the average behavior and nominal behavior of the mathematical model with 10 different sets of estimated rate constants. We also tested the robustness property of this model when the 10 sets of estimated rate constants were perturbed by the Gaussian random variable with strength 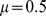 in equation (11). In this case the simulated perturbations of kinase activities are smaller than but still proportional to the corresponding perturbations in Figure 2A and 2B (results not shown). In addition, we tested the robustness property of the model using the 10 sets of the rejected rate constants that generated simulations with larger errors. Simulation results suggested that there is no correlation between the model estimation error and robustness property.

**Figure 3:**
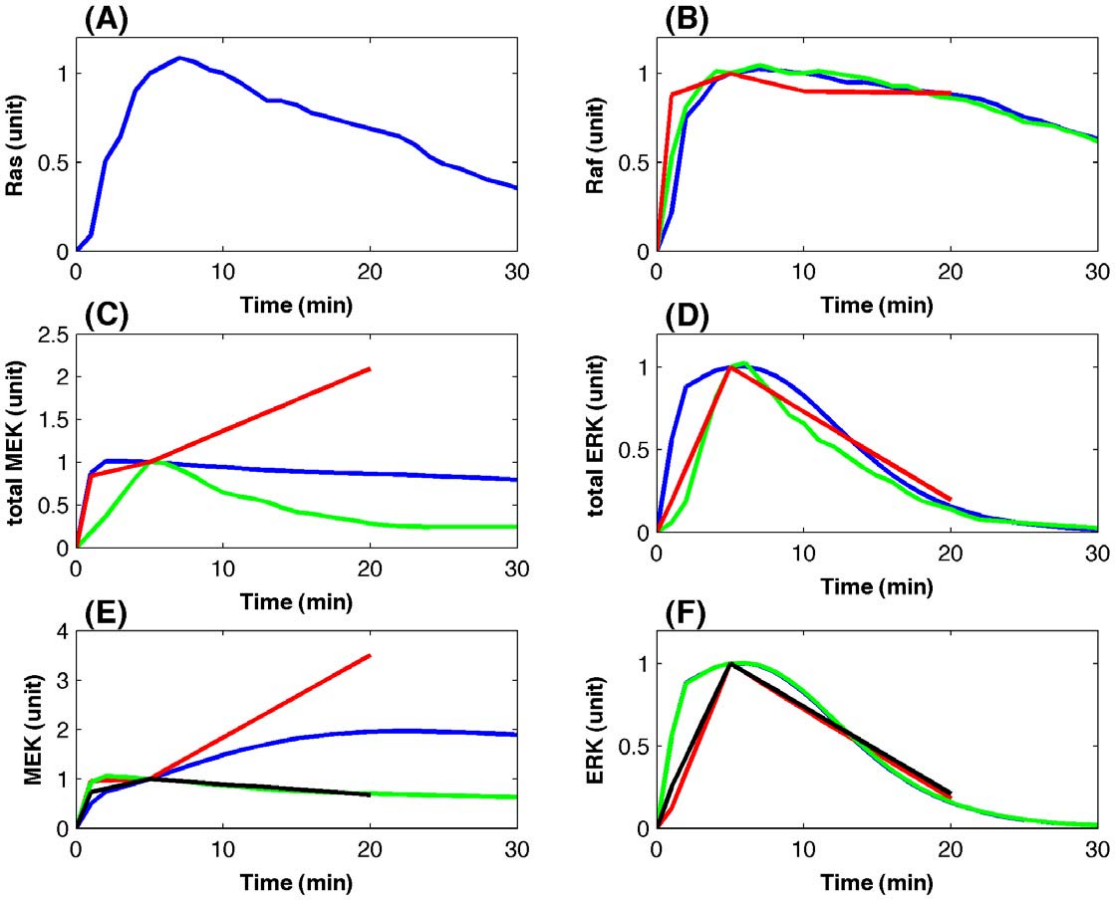
Simulations of the normalized kinase activities. (A) Normalized Ras activity as the signal input from [30]. (B) Raf activity; (C) Total MEK activity; and (D) Total ERK activity (blue-line: simulation; green-line: normalized Western blotting data [30]; red-line: proteomic data [9]). (E) MEK activity and (F) ERK activity at different locations (blue-line: simulation in the cytosol, red-line: proteomic data in the cytosol, green-line: simulation in the nucleus, black-line: proteomic data in the nucleus).

Figure 3 gives simulation results of the MAP kinase pathway using the model that has both small estimation error and good robustness property. The corresponding estimated model parameters were given in Table S1. To compare with the proteomic data, simulations were also normalized by the simulated kinase activity at 5 min. The total activity of MEK in Figure 3C (ERK in Figure 3D) was also normalized by the corresponding total kinase activity at 5 min. Simulations showed that the simulated kinase activities matched the Raf* activities in the cytosol (Figure 3B) and ERKpp activities in both the cytosol and nucleus (Figures 3F) quite well. In fact, the proteomic data of the normalized ERK activity in the cytosol are very close to those in the nucleus (Figure 3F). However, there is a large difference between the simulated MEK activities and proteomic data in Figure 3C. Note that there is a significant difference between the MEK kinase proteomic data in the cytosol and nucleus. The simulated MEK activities in the nucleus match the proteomic data very well. The derivation between the simulated MEK activities and proteomic data in the cytosol will be discussed in the next subsection.

To demonstrate the feasibility of our modeling approach, we compared our simulated kinase activities in Figure 3 with the kinase activities measured *in vivo* by Western blotting that were taken from Figure 7 in Ref [30]. To match the normalized proteomic data, the experimental activities of each kinase were also normalized by its activity at 5 min. Figure 3 shows that our computer simulation matched the Raf activity (Figure 3B) and ERK activity (Figure 3D) very well. The reason for the good agreement between the kinase activities is that the Western blotting data in [30] match the proteomic data very well. However, the measured MEK activity in Figure 3C is different from the proteomic data, and interestingly, the simulated MEK activity locates in the middle of the proteomic data and Western blotting data. Note that the simulated MEK activity is smaller, rather than being larger than the proteomic data, when time increases. The reason may be that, in order to match the ERK kinase activity that decreases significantly from 10 min to 20 min, MEK kinase activity should be smaller and smaller in this time period. This observation suggests that in the cell signaling cascade, the downstream signal activity may be used to calibrate the measurement errors of the upstream signals that are present in the proteomic datasets.

### Model refinement by incorporating more experimental data

Although the normalized simulation matches the proteomic data and experimental data very well in Figure 3, the robustness analysis results in Figure 2 suggested that the percentages of the activated kinases were quite low. In addition, the fraction of the activated MEK kinase was larger than that of the activated ERK, which is in contradiction to previous observations [26], [27], [30]. When using the absolute protein concentrations as the initial condition to simulate the established System 1, we found large difference between the predicted kinase activities and experimentally measured activities [30]. These results suggested that the normalized proteomic data might not be adequate for accurately inferring the cell signaling pathway. To achieve better inference results, it is clear that more experimental data should be incorporated to the model on the basis of the proteomic data [42].

To further refine the mathematical model, we used the experimentally measured absolute total concentration of each kinase, which were also the initial condition of System 2 in Table 1, together with the information regarding the maximal percentages of MEK and ERK kinases that were activated by EGF stimulation [30], which is also presented in Table 1. Then the normalized proteomic data (with kinase activity of unit one at 5 min) were rescaled by the absolute kinase activity (i.e. activated kinases at 5 min in System 2) in Table 1. The kinase activity in System 2 was calculated by
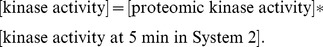
Note that the related activities of each kinase remained unchanged. In addition, the absolute concentrations of the three phosphatases, namely Raf-P'ase, MEK-P'ase and ERK-P'ase, were also included in the model using the experimentally measured data [26], [30], which is part of the initial condition of System 2 in Table 1. Note that the Raf, MEK and ERK kinase activities in Figure 7 in [30] were only utilized to compare with the simulated kinase activities, serving as the evidence to validate the feasibility of our proposed mathematical model. Since no further information was available regarding the distributions of the activated MEK and ERK kinases in different subcellular locations, we still used the proteomic data to generate the normalized kinase activities in the cytosol and nucleus. In summary, the experimental data provide: (1) the absolute concentrations of the activated Raf, total MEK activity and total ERK activity in the first 20 min stimulated by Ras-GTP-binding; (2) the normalized activities of MEK and ERK kinases in the cytosol and nucleus in the first 20 min.

We used these experimental data to infer the model rate constants once again. To balance the errors of different kinases, the weight to scale the errror of each kinase in equation (6) was the experimentally measured maximal activity of that kinase. However, for the normalized activities of MEK and ERK in the cytosol and nucleus, the weight in equation (6) was set to unit one. In this case we also derived 20 sets of estimated model rate constants by repeated implementations of the genetic algorithm and selected the top 10 sets with smaller estimation errors. For the top 10 sets of model rate constants, we used the same method described in the previous subsection to carry out the robustness analysis. Since the top 10 sets of estimates have similar kinetic fits, we selected the kinetic rates that produced the best robustness property of the system as our final estimate. The estimated model parameters were given in Table S1.

**Figure 4:**
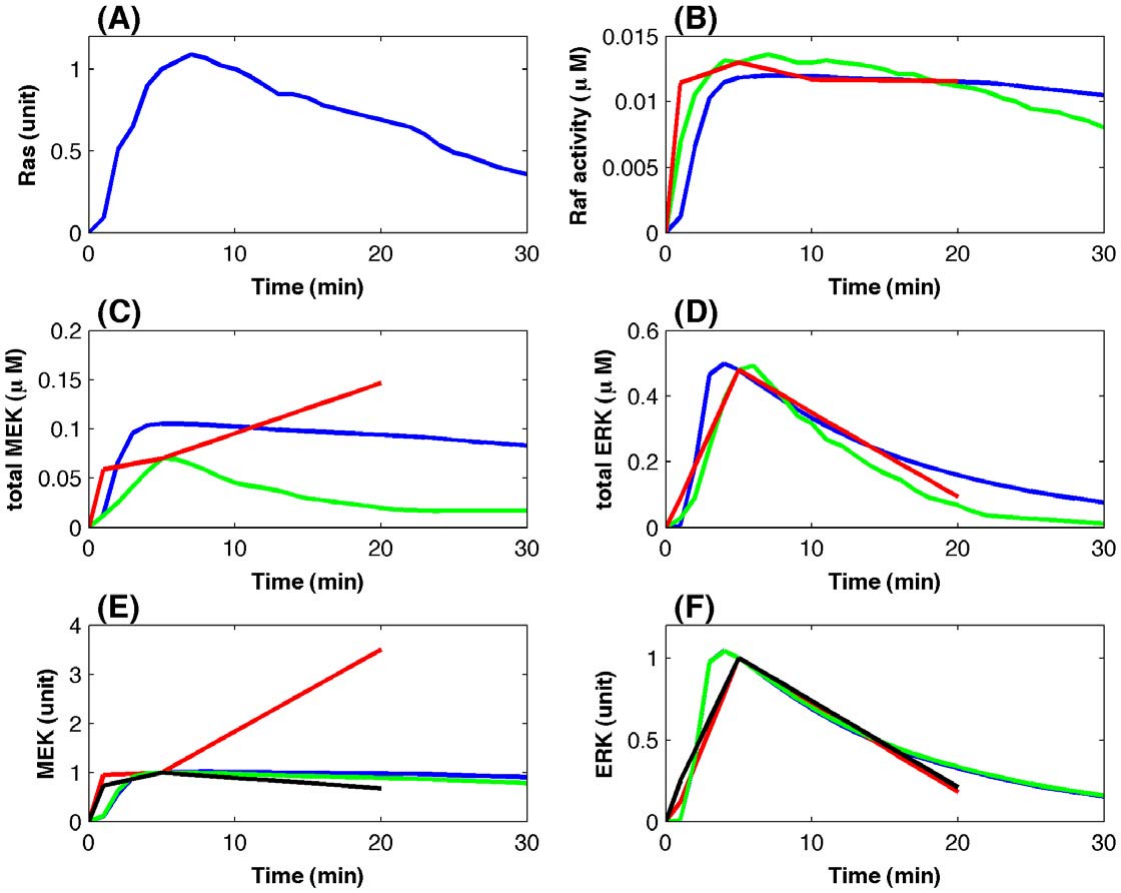
Simulated kinase activities based on the incorporation of proteomic data and Western blotting data. (A) Normalized Ras activity as the signal input [30]. (B) Raf activity; (C) Total MEK activity, and (D) Total ERK activity (blue-line: simulation; green-line: Western blotting data [30]; red-line: re-scaled proteomic data [9]). (E) MEK activity and (F) ERK activity at different locations (blue-line: simulation in the cytosol, red-line: proteomic data in the cytosol, green-line: simulation in the nucleus, black-line: proteomic data in the nucleus).

The major advantage of adding more experimental data is that the mathematical model now can realize experimental observations much more accurately and as a result computer simulations are able to make testable predictions regarding the regulatory mechanisms, which will be discussed in the following subsection. Figure 4 gives the simulated system dynamics with the absolute kinase activities. Computer simulations match the experimental data very well for the Raf activities in Figure 4B, and the total ERK kinase activities in Figure 4D. Moreover, the normalized MEK activity in the cytosol is very close to that in the nucleus, which is consistent with the experimental observation [30]. Compared with the simulations based on the normalized kinase concentrations in Figure 3, simulations using the absolute kinase concentrations in Figure 4 have better agreement with the experimental data.

An additional advantage of the refined model is that it has a very good robustness property in response to the perturbations in rate constants. Numerical results in Figure 2C and 2D suggested that the developed model based on the absolute kinase concentrations has a better robustness property than that based on the normalized kinase concentrations. Compared with the results in Figure 2A and 2B, variations between the kinase activities derived from perturbed and unperturbed rate constants in Figure 2C and 2D are much smaller. In particular, the variances of the Raf activity in Figure 2D are neglectable.

**Figure 5:**
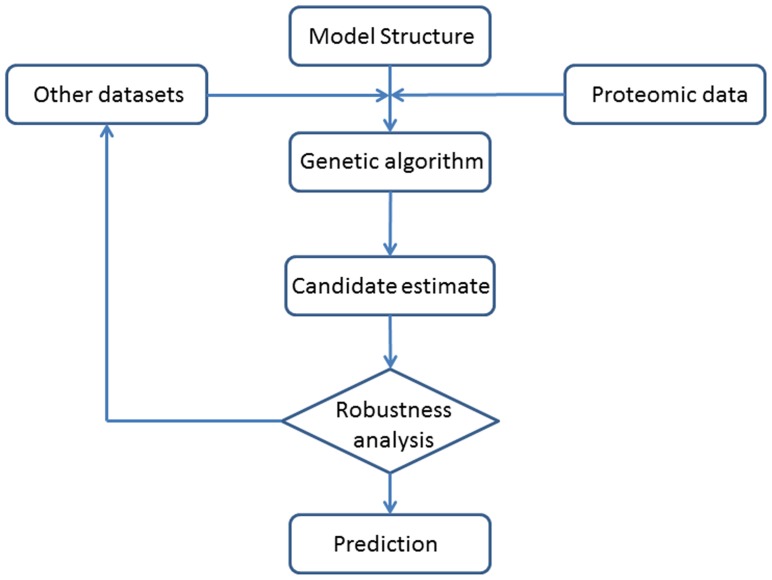
Flowchart of the proposed modeling framework for developing mathematical models of cell signaling pathways using proteomic data. Refer to the Section “Model refinement by incorporating more experimental data” for more detailed description of this flowchart.

In summary, the flowchart of the proposed modeling framework is given in Figure 5. The model structure may include the graphical schematic structure of the signaling pathway, a list of all the chemical reactions and a mathematical model that is a system of differential equations. The proteomic data are the time-course quantitative data of kinase activities. The other datasets include data resources obtained by other experimental techniques such as the FRET imaging and Western blotting. Using the genetic algorithm, we can obtain a number of candidate estimates of model parameters. It is suggested that the robustness analysis will only be applied to the selected candidates that have the minimal estimation errors in the genetic algorithm. If the robustness analysis results are satisfactory, we can choose the parameter set that has the best robustness property as our final parameter estimate. Otherwise, other experimental data are required to refine the parameter estimation. Finally, we can make testable predictions regarding the signal output under various system conditions.

### Signal output inhibited by phosphatases with different concentrations

To further validate the feasibility of our modeling approach, we next asked whether the developed model could make testable predictions. Here we used the MAP kinase pathway inhibited by phosphatases with different concentrations as a test problem to predict the signal output under different cellular conditions. This test has important implications for drug design because protein phosphatases are viable therapeutic targets [43], [44]. The activity of protein phosphatases can be manipulated to alter cellular signaling for therapeutic benefits [44]. In this work we tested the effects of two important phosphatases PP2A and MKP3. It has been well established that MKP3 is a phosphatase to inhibit the activities of both MEK and ERK [45]. However, the regulatory function of PP2A in the MAP kinase pathway is complex. PP2A positively promotes Raf kinase activation by dephosphorylating activated Raf and scaffold protein KSR, regulating 14-3-3 interactions and stimulating the recruitment of Raf and KSR complex to the plasma membrane [46], [47], [48], [49], [50]. Simultaneously, as a phosphatase, PP2A inhibits ERK pathway by dephosphorylating the activated MEK kinase and is involved in the regulation of nearly all cellular activities [27], [51], [52]. Therefore, different PP2A activities were realized by different concentrations of MEK phosphatase (denoted as MEK-P'ase). In addition, since scaffold protein KSR was not included in our proposed model, the positive regulation of PP2A was described by the PP2A dependent binding rate 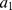
of Ras and Raf, given by
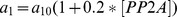
where 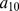 is the basal binding rates. In addition, different MKP3 activities were implemented by different concentrations of MEK-P'ase and ERK phosphatase ERK-P'ase. Although ∼25% of ERK phosphatases are assumed to be serine/threonine phosphatases and thus are able to dephosphorylate MEKp and MEKpp, it should be noted, however, that only a fraction of the ERK phosphatases can deactivate the activated MEK kinase [27]. Therefore we assumed that only a quarter of MEK-P'ase varied proportionally to the MKP3 concentrations.

**Figure 6:**
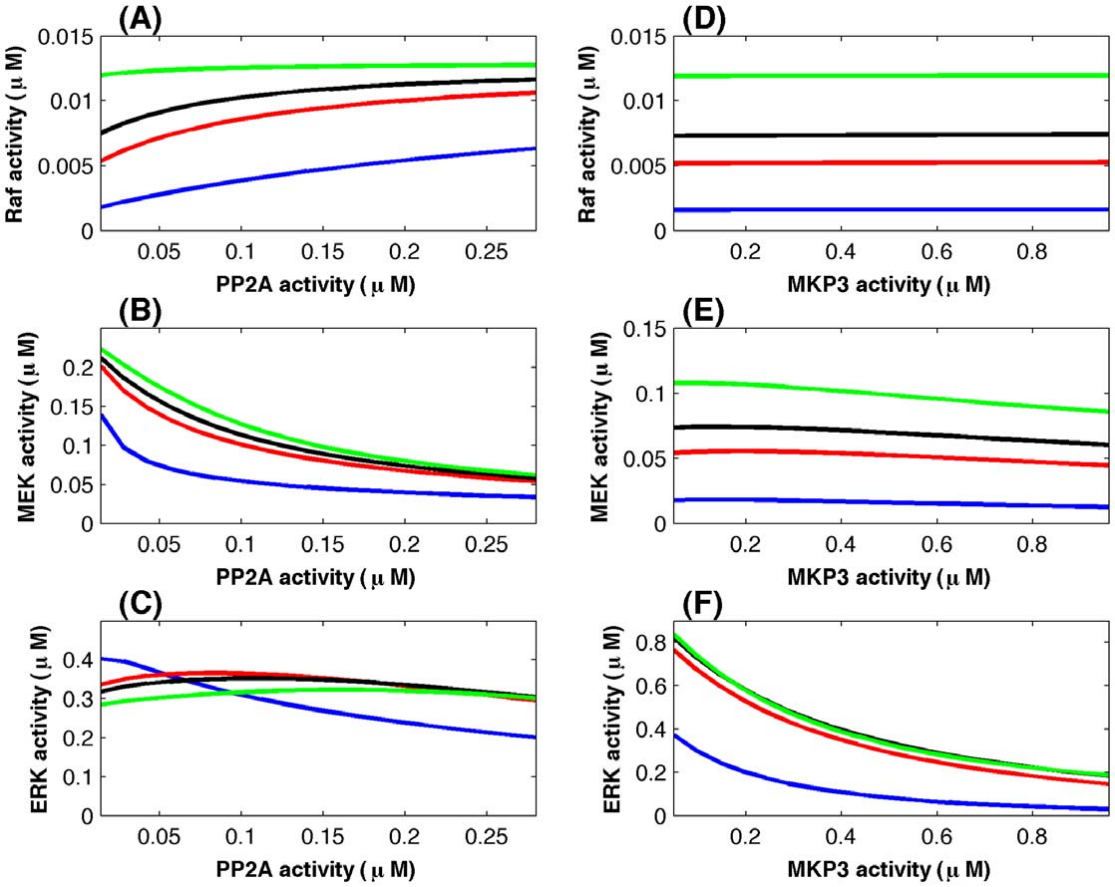
Kinase activities at 10 min inhibited by phosphatases PP2A and MKP3. (A, B, C) Simulated Raf, MEK and ERK activities at 10 min when the MAP kinase module was stimulated by different signal inputs and inhibited by phosphatase PP2A with different concentrations. (D, E, F) Simulated Raf, MEK and ERK activities at 10 min when the MAP kinase module was stimulated by different signal inputs and inhibited by the phosphatase MKP3 with different concentrations (blue-line: Ras = 0.004; red-line: Ras = 0.02; black-line: Ras = 0.04; green-line: Ras = 0.4).

According to the experimental conditions in [45], we used the mathematical model with the absolute kinase concentrations to simulate kinase activities, when the MAP kinase module was stimulated by Ras-GTP with activities ranging from 0.004 to 0.4 and the scaled phosphatase concentrations ranging from 0.3 to 2, respectively. Figure 6 shows that the simulated kinase activities at 10 min are in good agreement with the experimental data [45]. Since the MEK and ERK activities begin to decline from ∼6 min, the different measurement points may lead to different patterns of the kinase activities in regards to different ligand concentrations and phosphatase activities. The predicted kinase activities measured at 5 min or 20 min are given in the Figure S1 and Figure S2, respectively. The simulated kinase activities at 10 min and 20 min suggested that the positive regulation of Raf activation by PP2A is important to maintain a sustained MAP kinase activity over the time course.

## Discussion

In this work, we used a well-characterized pathway to develop a general framework to design mathematical models of cell signaling pathways based on proteomic datasets. The power of this modeling framework lies in its potential ability to explore the network structure of more complex signaling pathways. Recently systems-wide studies of targeted biochemical assays and *in vivo* phosphoproteome have identified thousands of *in vivo* phosphorylation sites in signaling proteins across different biological conditions [1], [6]. These achievements have led to a significantly accelerating expansion of our knowledge regarding the kinase-substrate relationships and post-translational modifications in cell signaling pathways. However, our understanding of the phosphorylation-dependent signaling cascades is far from complete. In this context, the motivation to better understand the essential mechanisms underlying signaling pathways has driven the development of bioinformatic and systems biology approaches to infer signaling networks by exploiting high-throughput genomic and/or proteomic data [53], [54], [55], [56]. Development of such methods for inferring signaling networks will significantly enhance our ability to accurately model signaling networks and to discover new mediators or components of the known networks. These bioinformatics tools will be used in our modeling framework as a crucial first step to infer the structure of signaling cascades.

In spite of the significant progress in the phosphoproteomics by using mass spectrometry, this technique also has certain limitations. Among them, the lack of information on the stoichiometry of phosphorylation is the key limitation of the current phosphoproteomic approaches [57]. In addition, quantification may be limited to a portion of proteins (for example, proteins with adequate abundance) [58], which leads to the missing values or the absence of relevant proteins. Another limitation is the resolution of time-course studies, so certain fast phosphorylation events may be very difficult to be picked up experimentally [59]. It is worth noting that these limitations may be liable to errors in the proteomic data. Even by making the most out of the available data, this research work has identified a number of important issues in using proteomic data to infer mathematical models of cell signaling pathways. One of the challenging issues is the missing value of kinase activities, which can be caused by either biological or technical reasons. Although a number of statistical methods have been proposed to estimate the missing value, the implementation of these methods will be extremely difficult if the activity of a protein is completely unavailable in the proteomics dataset. An example is the Ras protein whose activities were not available in the proteomic dataset at all. In this case, other sources of biological data must be utilized to fill the data gaps. In addition, the normalization of proteomic data causes the uncertainty of protein concentrations in mathematical modeling. In this work we first used the unified protein concentrations, where the information of the absolute protein concentrations was not known *a priori*. Although the normalized simulations can match the normalized experimental data very well, the simulated relative protein concentrations do not necessarily reflect the real scenario of signaling pathways, since the concentrations of proteins, such as MEK-P'ase [27], may play an appreciated role in modulating signaling transduction. Therefore, it is important to enrich the data by integrating more sources of experimental data prior to model simulation. In fact, by incorporating more relevant information regarding the absolute kinase concentration and percentages of activated kinases in this study, we have shown that the developed mathematical model has indeed achieved a better simulation accuracy and more robust properties with respect to varying rate constants. More importantly, the mathematical model can provide more realistic predictions and mechanistic insights into kinase activities under various cellular conditions.

The MAP kinase module studied in this work comprises of only seven proteins. Based on the detailed phosphorylation and dephosphorylation reactions, the proposed mathematical model encompasses 33 reactions and 57 unknown rate constants. By using only a small amount of proteomic data that are currently available to infer a large number of rate constants, our research showed that a variety of rate constants could reliably realize the same experimental data. Thus a challenging question is how to select the most appropriate rate constants from a variety of candidate estimates. One possible approach to address this issue is to develop simplified mathematical models with less unknown rate constants, which is similar to the approaches to infer gene regulation from microarray data [60], [61]. The Michaelis-Menten function is one of the best known models to simplify the enzymatic reactions and reduce the number of unknown parameters. However, the remaining question in the field is how to design simplified functions to represent the multiple-step activation and deactivation reactions which are essential for signaling transduction. In addition, we need to develop useful methods to identify the key steps of phosphorylation or dephosphorylation reactions.

Proteomics data are subjected to considerable noise, including not only the technical noise arising from repeated experimental processes but also the analysis noise [62]. However, compared with the developed stochastic methods for studying noise in microarray expression data [63], [64], the study of noise in proteomic data is still at the very early stage of development. Researchers are still in the progress of designing noise models to characterize the statistical distributions of noise in proteomic data. Noise, like the error of MEK kinase activity in this study, may result in significant variations in the inference of mathematical models. However, an interesting observation in this study is that the downstream signal cascade may have the potential to correct the errors in the upstream signal activity. In view of this, more work is required to investigate the influence of noise on the development of mathematical models based on the noisy proteomic datasets.

The MAP kinase pathway is one of the most extensively studied signalling pathways. Over the last two decades there has been a large amount of published experimental data regarding the signalling entities, regulatory interactions, kinase activities, protein absolute concentrations and perturbation studies. In addition, the advances in systems biology have produced a number of sophisticated mathematical models with various assumptions of the regulatory mechanisms at different levels as well as inferred model parameters from experimental data under various experimental conditions and from different types of cells. In this work we concentrated on the issue of establishing mathematical models from proteomic datasets. However, only a small amount of experimental data was utilized in this work to refine the developed mathematical model. As a result, our simulation suggested that the integration of more experimental data could improve the accuracy of the mathematical model substantially. Therefore, the future work includes the development of more sophisticated models for cell signalling pathways based on the combination of large-scale proteomic datasets, more experimental data, more signalling regulatory mechanisms as well as estimated model parameters.

In summary, this work proposed a novel computational framework to develop mathematical models of cell signaling pathways based on the proteomic datasets. Using the MAP kinase pathway as the test system, we developed a new mathematical model including the cytosolic and nuclear subsystems and applied the genetic algorithm to infer unknown model parameters. The robustness property of the mathematical model was used as a criterion to select the appropriate rate constants from the estimated candidates. This research work identified a number of important issues in using proteomic data to infer the cell signaling pathways. Our results have also demonstrated that incorporation of more relevant experimental data, including the absolute protein concentrations, could significantly enhance not only the simulation accuracy but also the robustness property of the proposed mathematical model. The successful application of the proposed modelling framework to the MAP kinase pathway suggests that this approach is very promising for developing accurate and robust mathematical models for more complex cell signaling pathways.

## Materials and Methods

### Experimental data

Using an integrated phosphoproteomic technology that combines phosphopeptide enrichment, high-accuracy identification, and stable isotope labeling by amino acids in cell culture (SILAC) with the time-course method, Olsen et al. [9] have recently identified and quantitated the global *in vivo* phosphoproteome and its temporal dynamics upon growth-factor stimulation in human HeLa cells. In this study, human Hela cells were stimulated with 150 ng/ml of EGF for different time periods. The temporal dynamical profiles were recorded in the Phosida database [9]. This dataset includes the quantitative temporal activity ratios of 2,244 proteins with a total of 6,600 phosphorylation sites, and can be download as an excel file in the supplementary information of ref [9]. Note that the computational approach for extracting quantitative proteomic data from proteomic readout is a crucial step in proteomic data analysis and mathematical modeling. Here we referred to two review papers [65], [66] and the further references therein for the recent progress of the experimental and computational approaches for extracting proteomic data.

We used the proteomic data of the ARaf1 protein, the dual specificity mitogen-activated protein kinase kinase 2 (MEK) and the mitogen-activated protein kinase 1 (ERK) in Table S1. In this dataset kinase activities were measured at 0, 1, 5, 10 and 20 min. The activities of each kinase were normalized by its activity at 5 min. The activities of ARaf1 were available in the cytosol only; while the activities of MEK and ERK were obtained in both the cytosol and nucleus.

Additional experimental data were also obtained by using Western blotting analysis and other experimental techniques in human HeLa cells [30]. Hela cells were stimulated with 50 ng/ml of EGF for different time periods. Although the EGF concentration in this study is different from that in the study [9], it has been indicated that the proportion of phosphorylated MEK remained unchanged even in the presence of an excess of EGF [30]. Therefore both datasets in the studies [9], [30] can be perfectly combined in our study. The Ras activity in Figure 7 [30] was used as the signal input of the MAP kinase module in this research. We also used the absolute kinase concentrations in Table 1 in [30] and the fractions of the activated kinases (at 5 min) in Figure 2 in [30] in our modeling work, which led to the absolute activated kinase concentrations at 5 min in Table 1 in this paper. Then the relative kinase activities in the proteomic study were re-scaled by the absolute activated kinase concentrations at 5 min. Note that the Raf, MEK and ERK kinase activities in Figure 7 [30] were utilized only to compare with the simulated kinase activities, serving as an evidence to validate the feasibility of our proposed mathematical model.

In addition, the kinase activities that were inhibited by different phosphatases [45] were also used to validate the predictions derived from our proposed model.

### Inference method

All model parameters are estimated by using the genetic algorithm, which is an effective searching method for finding the unknown kinetic rates when the search space is associated with a complex error landscape. We used a MATLAB toolbox developed by Chipperfield et al. [67] to infer the 57 unknown rate constants. This toolbox used MATLAB functions to build a set of versatile routines for implementing a wide range of genetic algorithms. The major procedures of the genetic algorithm toolbox include population representation and initiation, fitness assignment, selection functions, crossover operators, mutation operators and multiple subpopulation support. In this work we used the function *crtbp* to create the binary initial population, the linear-ranking and non-linear-ranking algorithms *ranking* to transform the raw objective function values into non-negative figures of merit for each individual, a selection function *reins* to effect fitness-based reinsertion when the entire population is not reproduced in each generation, a high-level entry function *select* to provide a convenient interface to the selection routines, a high-level entry function *recombine* to provide all the crossover operators, and the routine *mut* to perform binary and integer mutations.

The genetic algorithm was run over 500 generations for each rate estimate, and we used a population of 100 individuals in each generation. The values of rate constants were taken initially from the uniform distribution in the range of [0,W_max_], and the value of W_max_ was fixed to 1000 for each rate constant. The initial estimate of rate constants can be changed by using different random seeds in the MATLAB toolbox, leading to different final estimates of the rate constants.

The estimation error was measured by the weighted distance between the simulated kinase activities and experimental data [68]. The weight of each kinase was determined by its corresponding maximal activity. The total error is calculated by
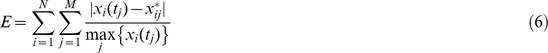
where 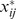 and 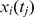 are the simulated and experimentally measured activities of kinase 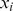 at time point 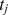, respectively. Note that in the “Estimation of model kinetic rates” section 
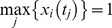
. However, in the “Model refinement” section, the total concentration of each kinase was scaled by its maximal activity; but for the MEK and ERK activities in different cellular locations,
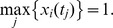



### Robustness analysis

We used the concept defined by Kitano [34] to measure the robustness property of the proposed model. The robustness property of a mathematical model with respect to a set of perturbations 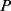 is defined as the average of an evaluation function 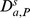 of the system over all perturbations 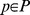, weighted by the perturbation probabilities 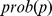, given by
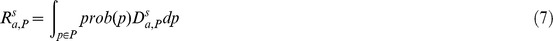
Here we proposed to use the following measure to evaluate the average behavior
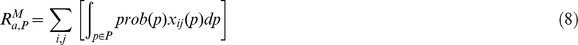
which is the mean of kinase activities that should be close to the simulated kinase activity obtained from the unperturbed rate constants. In addition, the impact of perturbations on nominal behaviour is defined by
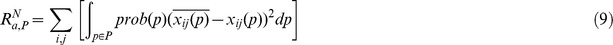
where 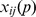 and 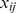 are the simulated activities of kinase 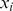 at time point 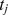 with perturbed and unperturbed rate constants, respectively, and 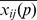 is the mean of 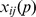 over all the perturbated kinetic rates.

For each rate constant 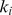, the perturbation is set to
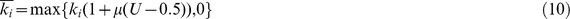
with a uniformly distributed random variable *U(0,1)* or
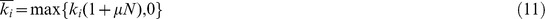
with the standard Gaussian random variable *N(0,1)*. Here 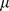 represents the perturbation strength.

## Supporting Information

Figure S1 **Kinase activities at 5 min inhibited by phosphatases PP2A and MKP3.** (A, B, C) Simulated Raf, MEK and ERK activities at 5 min when the MAP kinase module was stimulated by different signal inputs and inhibited by phosphatase PP2A with different concentrations. (D, E, F) Simulated Raf, MEK and ERK activities at 5 min when the MAP kinase module was stimulated by different signal inputs and inhibited by the phosphatase MKP3 with different concentrations (blue-line: Ras = 0.004; red-line: Ras = 0.02; black-line: Ras = 0.04; green-line: Ras = 0.4).
(TIF)

Figure S2 **Kinase activities at 20 min inhibited by phosphatases PP2A and MKP3.** (A, B, C) Simulated Raf, MEK and ERK activities at 20 min when the MAP kinase module was stimulated by different signal inputs and inhibited by phosphatase PP2A with different concentrations. (D, E, F) Simulated Raf, MEK and ERK activities at 20 min when the MAP kinase module was stimulated by different signal inputs and inhibited by the phosphatase MKP3 with different concentrations (blue-line: Ras = 0.004; red-line: Ras = 0.02; black-line: Ras = 0.04; green-line: Ras = 0.4).
(TIF)

Table S1 **Model kinetic rates.**
(DOCX)

Supporting Information S1 Section 1. Chemical reactions. Section 2. Mathematical model.
(DOCX)
