# Supplementary figures and images for: Mathematical Modelling of the MAP Kinase Pathway Using Proteomic Datasets
Source: PLoS One. 2012 Aug 8;7(8):e42230. doi: 10.1371/journal.pone.0042230 (PMC3414524; doi:10.1371/journal.pone.0042230)

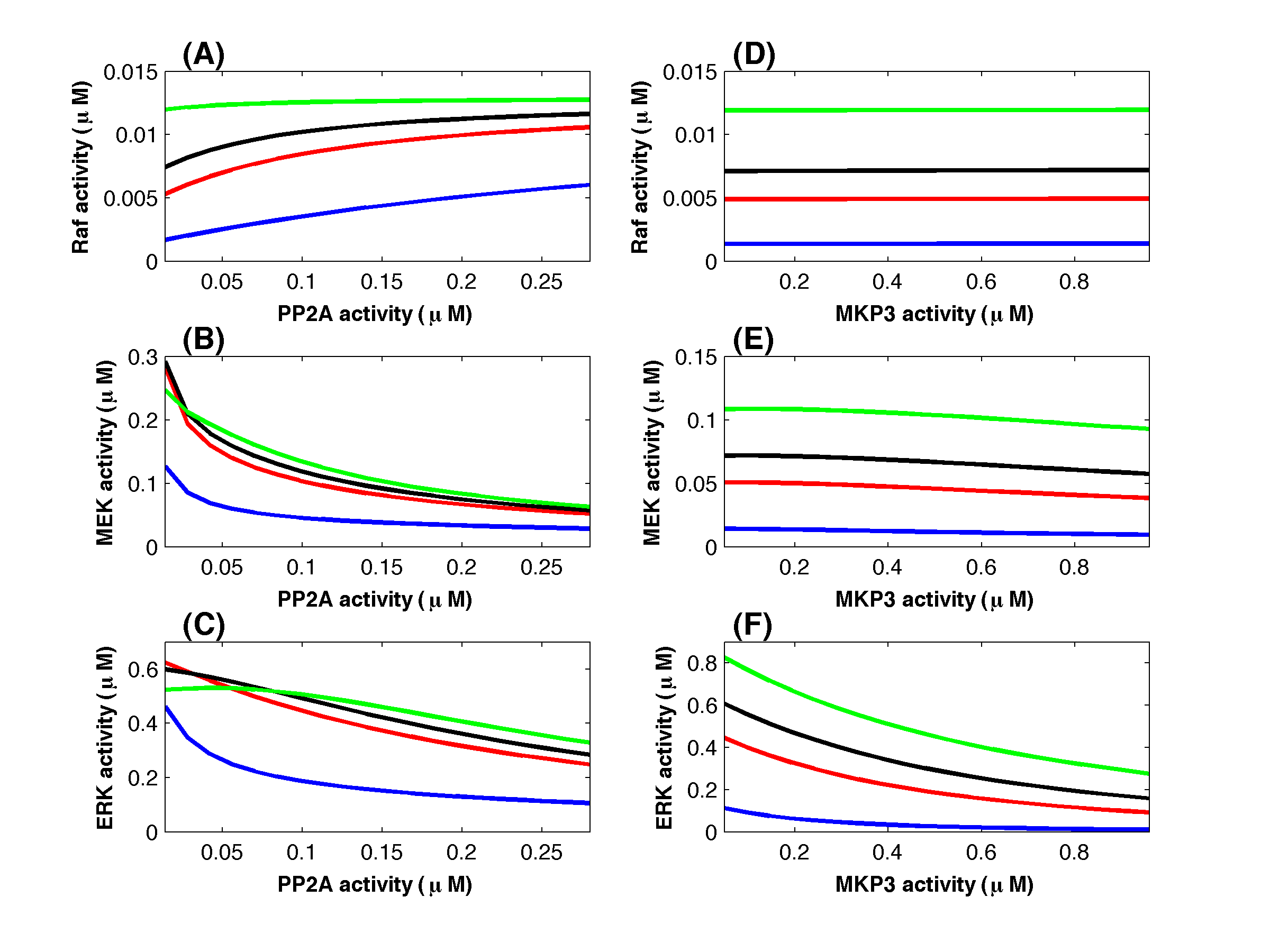

Supplement: Figure S1 — Kinase activities at 5 min inhibited by phosphatases PP2A and MKP3. (A, B, C) Simulated Raf, MEK and ERK activities at 5 min when the MAP kinase module was stimulated by different signal inputs and inhibited by phosphatase PP2A with different concentrations. (D, E, F) Simulated Raf, MEK and ERK activities at 5 min when the MAP kinase module was stimulated by different signal inputs and inhibited by the phosphatase MKP3 with different concentrations (blue-line: Ras = 0.004; red-line: Ras = 0.02; black-line: Ras = 0.04; green-line: Ras = 0.4). (TIF) [file pone.0042230.s001.tif]

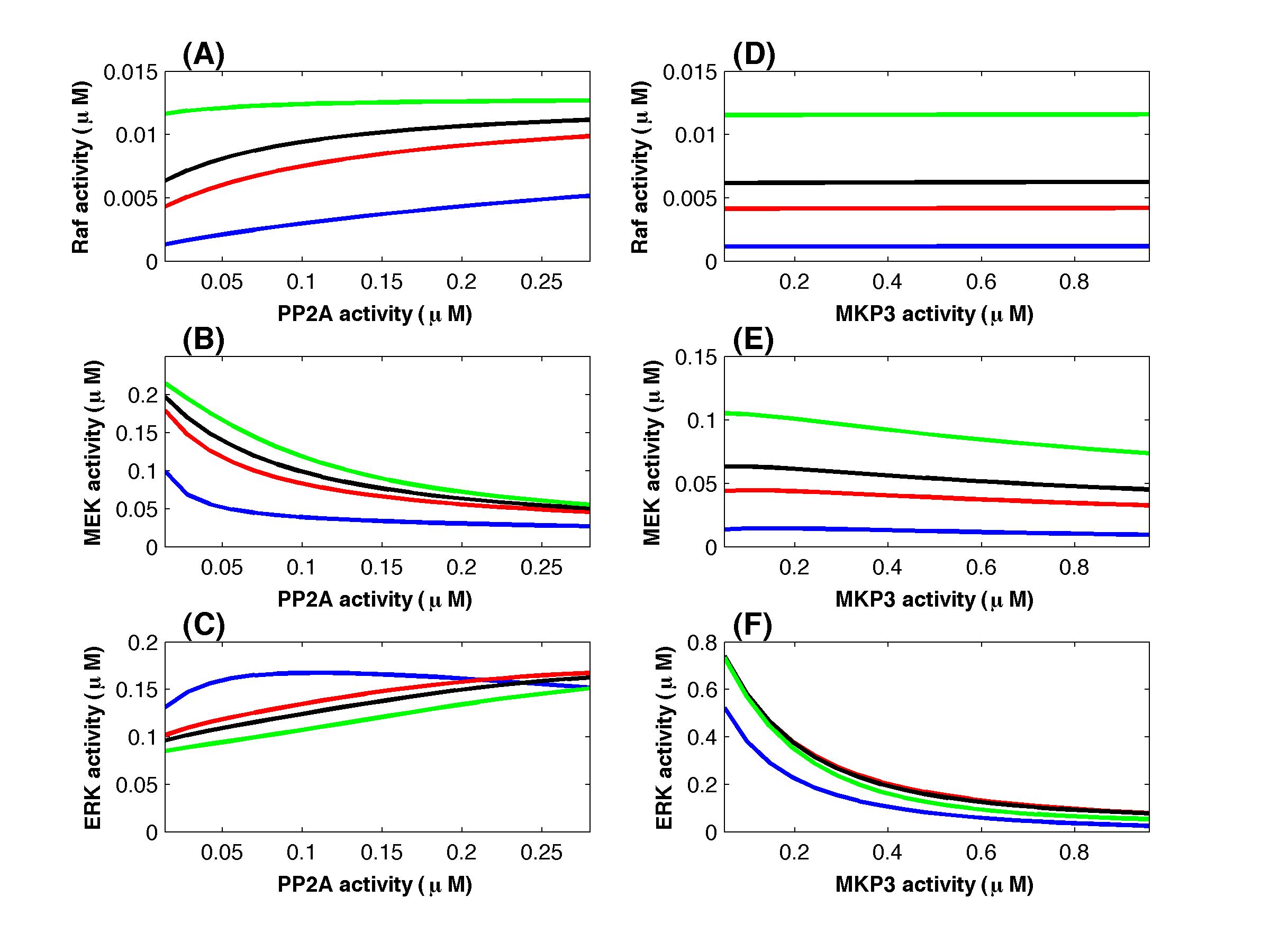

Supplement: Figure S2 — Kinase activities at 20 min inhibited by phosphatases PP2A and MKP3. (A, B, C) Simulated Raf, MEK and ERK activities at 20 min when the MAP kinase module was stimulated by different signal inputs and inhibited by phosphatase PP2A with different concentrations. (D, E, F) Simulated Raf, MEK and ERK activities at 20 min when the MAP kinase module was stimulated by different signal inputs and inhibited by the phosphatase MKP3 with different concentrations (blue-line: Ras = 0.004; red-line: Ras = 0.02; black-line: Ras = 0.04; green-line: Ras = 0.4). (TIF) [file pone.0042230.s002.tif]
