# Supplementary material for: Mathematical Modelling of the MAP Kinase Pathway Using Proteomic Datasets
Source: PLoS One. 2012 Aug 8;7(8):e42230. doi: 10.1371/journal.pone.0042230 (PMC3414524; doi:10.1371/journal.pone.0042230)
Supplement: Table S1 — Model kinetic rates. (DOCX) [file pone.0042230.s003.docx]

**Section 3 Model parameters**

**Supplementary Table 1: Model kinetic rates**

|  | System 1 (with normalized protein concentrations) | Systems 2 (with absolute protein concentrations) |
| --- | --- | --- |
| a1 | 627.9894 | 12.9004 |
| a2 | 130.0689 | 296.9649 |
| a3 | 771.4746 | 787.5564 |
| a4 | 991.0612 | 962.5454 |
| a5 | 392.3954 | 117.0922 |
| a6 | 178.0273 | 8.1720 |
| a7 | 303.2468 | 110.5682 |
| a8 | 990.7646 | 22.9655 |
| a9 | 262.2502 | 50.1080 |
| a10 | 30.7684 | 413.9485 |
| a11 | 258.6854 | 578.0903 |
| a12 | 421.6418 | 545.6148 |
| a13 | 934.5998 | 597.8275 |
| a14 | 997.4956 | 715.5311 |
| a15 | 308.2011 | 950.0722 |
| d1 | 966.7549 | 829.0270 |
| d2 | 545.3449 | 269.5801 |
| d3 | 311.4579 | 931.6711 |
| d4 | 81.7033 | 411.4126 |
| d5 | 555.8324 | 461.8659 |
| d6 | 771.3258 | 992.0931 |
| d7 | 868.4128 | 941.7581 |
| d8 | 805.8842 | 542.6078 |
| d9 | 848.7862 | 985.8656 |
| d10 | 616.4328 | 228.2631 |
| d11 | 525.1088 | 683.4208 |
| d12 | 3.5992 | 359.6071 |
| d13 | 648.7319 | 984.9748 |
| d14 | 0.5312 | 310.1609 |
| d15 | 991.2510 | 876.6049 |
| k1 | 378.9429 | 673.1917 |
| k2 | 954.3705 | 347.7281 |
| k3 | 999.2599 | 527.5817 |
| k4 | 711.0431 | 94.4081 |
| k5 | 142.5897 | 966.8493 |
| k6 | 807.5169 | 905.9800 |
| k6 | 202.6541 | 683.1633 |
| k7 | 984.7507 | 860.5174 |
| k9 | 487.7677 | 776.3679 |
| k10 | 105.4593 | 150.1161 |
| k11 | 35.2583 | 486.3171 |
| l12 | 979.5608 | 415.2683 |
| k13 | 142.6479 | 17.8394 |
| k14 | 41.8387 | 625.4545 |
| k15 | 70.9315 | 450.5782 |
| f1 | 359.1159 | 856.2792 |
| f2 | 60.0072 | 981.8249 |
| f3 | 141.0533 | 549.4905 |
| f4 | 15.8959 | 97.4713 |
| f5 | 141.4272 | 5.0669 |
| f6 | 486.5250 | 146.6915 |
| f7 | 328.0910 | 34.3962 |
| f8 | 669.2549 | 858.4803 |
| f9 | 282.8181 | 897.4122 |
| f10 | 955.9907 | 453.4173 |
| f11 | 516.0594 | 951.8842 |
| f12 | 2.2888 | 7.1974 |
