# Supplementary material for: Mathematical Modelling of the MAP Kinase Pathway Using Proteomic Datasets
Source: PLoS One. 2012 Aug 8;7(8):e42230. doi: 10.1371/journal.pone.0042230 (PMC3414524; doi:10.1371/journal.pone.0042230)
Supplement: Supporting Information S1 — Section 1. Chemical reactions. Section 2. Mathematical model. (DOCX) [file pone.0042230.s004.docx]

**Supplementary Information**

**Mathematical modelling of the MAP kinase pathway using proteomic datasets**

**Tianhai Tian and Jiangning Song**

**Section 1. Chemical reactions**

All the chemical reactions for the activation and dephosphorylation in the MAP kinase pathway are listed below. Kinase MEK is denoted for kinase in the cytosol, while N-MEK is located in the nucleus.

Ras + Raf $\begin{matrix} \xrightarrow{a1} \\ \xleftarrow[d1]{} \end{matrix}$ Ras-Raf $\xrightarrow{k1}$ Ras + Raf*

Raf* + Raf-P’ase $\begin{matrix} \xrightarrow{a2} \\ \xleftarrow[d2]{} \end{matrix}$Raf*- Raf-P’ase $\xrightarrow{k2}$Raf + Raf-P’ase

Raf* + MEK $\begin{matrix} \xrightarrow{a3} \\ \xleftarrow[d3]{} \end{matrix}$Raf*-MEK $\xrightarrow{k3}$ Raf* + MEKpp

MEKp+MEK-P’ase $\begin{matrix} \xrightarrow{a4} \\ \xleftarrow[d4]{} \end{matrix}$MEKp- MEK-P’ase $\xrightarrow{k4}$MEK + MEK-P’ase

MEKpp+ MEK-P’ase $\begin{matrix} \xrightarrow{a5} \\ \xleftarrow[d5]{} \end{matrix}$MEKpp- MEK-P’ase $\xrightarrow{k5}$ MEKp + MEK-P’ase

MEKpp+ERK$\begin{matrix} \xrightarrow{a6} \\ \xleftarrow[d6]{} \end{matrix}$MEKpp-ERK $\xrightarrow{k6}$MEKpp + ERKp

MEKpp+ERKp $\begin{matrix} \xrightarrow{a7} \\ \xleftarrow[d7]{} \end{matrix}$MEKpp-ERKp $\xrightarrow{k7}$MEKpp + ERKpp

ERKp+ ERK-P’ase $\begin{matrix} \xrightarrow{a8} \\ \xleftarrow[d8]{} \end{matrix}$ERKp- ERK-P’ase $\xrightarrow{k8}$ERK + ERK-P’ase

ERKpp+ ERK-P’ase $\begin{matrix} \xrightarrow{a9} \\ \xleftarrow[d9]{} \end{matrix}$ERKpp- ERK-P’ase $\xrightarrow{k9}$ERKp + ERK-P’ase

N-MEKp+ MEK-P’ase $\begin{matrix} \xrightarrow{a10} \\ \xleftarrow[d10]{} \end{matrix}$N-MEKp- MEK-P’ase $\xrightarrow{k10}$N-MEK + MEK-P’ase

N-MEKpp+ MEK-P’ase $\begin{matrix} \xrightarrow{a11} \\ \xleftarrow[d11]{} \end{matrix}$N-MEKpp- MEK-P’ase $\xrightarrow{k11}$ N-MEKp + MEK-P’ase

N-ERKp+ ERK-P’ase $\begin{matrix} \xrightarrow{a12} \\ \xleftarrow[d12]{} \end{matrix}$N-ERKp- ERK-P’ase $\xrightarrow{k12}$N-ERK + ERK-P’ase

N-ERKpp+ ERK-P’ase $\begin{matrix} \xrightarrow{a13} \\ \xleftarrow[d13]{} \end{matrix}$N-ERKpp- ERK-P’ase $\xrightarrow{k13}$N-ERKp + ERK-P’ase

N-MEKpp+N-ERK $\begin{matrix} \xrightarrow{a14} \\ \xleftarrow[d14]{} \end{matrix}$ N-MEKpp-N-ERK $\xrightarrow{k15}$ N-MEKpp + N-ERKp

N-MEKpp+N-ERKp $\begin{matrix} \xrightarrow{a15} \\ \xleftarrow[d15]{} \end{matrix}$ N-MEKpp-N-ERKp $\xrightarrow{k15}$ N-MEKpp + N-ERKpp

MEKpp $\xrightarrow{f1}$N-MEKpp

ERKpp $\xrightarrow{f2}$ N-ERKpp

N-MEK $\xrightarrow{f3}$ MEK

N-ERK $\xrightarrow{f4}$ ERK

N-MEKpp $\xrightarrow{f5}$ MEKpp

N-ERKpp $\xrightarrow{f6}$ ERKpp

MEK $\xrightarrow{f7}$ N-MEK

ERK $\xrightarrow{f8}$ N-ERK

MEKp $\xrightarrow{f9}$ N-MEKp

N-MEKp $\xrightarrow{f10}$ MEKp

ERKp $\xrightarrow{f11}$ N-ERKp

N-ERKp $\xrightarrow{f12}$ ERKp

**Section 2. Mathematical model**

Based on the chemical reactions listed in Section 1, we developed a mathematical model of differential equations given by
